# Full-length transcriptome analysis of papillary thyroid carcinoma reveals correlation between *LAMB3* expression and clinical features

**DOI:** 10.1186/s12885-025-14916-0

**Published:** 2025-10-25

**Authors:** Shang Lyu, Yadi Wang, Fang Chai, Jia Zhang, Nan Zhang, Congying Zhao, Zimeng Song, Lina Feng, Jing Zhang, Yue Xi

**Affiliations:** 1 Jinzhou Medical University, Jinzhou, China; 2 Department of Endocrinology, The Third Affiliated Hospital of Jinzhou Medical University, Jinzhou, China; 3 Thyroid Surgery Department, The First Affiliated Hospital of Jinzhou Medical University, Jinzhou, China; 4 Changchun Infection Disease Hospital, Changchun, China; 5 Shenbei New District Center for Disease Control and Prevention of Shenyang (Shenbei New District Health Supervision Institute of Shenyang), Shenyang, China

**Keywords:** Papillary thyroid carcinoma, Third-generation sequencing, *LAMB3*, Biomarker, Metastasis

## Abstract

**Background:**

Thyroid carcinoma is the most common malignant endocrine tumour, and its prevalence has been on the rise in recent years. However, mechanisms underlying the metastasis of thyroid carcinoma and candidate biomarkers remain elusive. In this study, we screened genes involved in the virulence and metastasis of papillary thyroid carcinoma (PTC).

**Methods:**

Oxford Nanopore Technology full-length transcriptome sequencing and bioinformatic analyses were performed to analyse differentially expressed genes (DEGs) in PTC. We collected 15 cancerous and paracancerous tissue pairs from patients with PTC for RNA sequencing. The significance thresholds for DEGs were |log2(fold change)| ≥ 1 and false discovery rate (FDR) < 0.01. Gene Ontology and Kyoto Encyclopaedia Gene and Genome pathway enrichment analyses of the 50 most significant DEGs were performed. Immunohistochemistry was used to evaluate *LAMB3* expression in tissue microarrays (58 pairs of PTC samples) and its correlation with clinicopathological parameters. Differences in gene expression between cancerous and paracancerous tissues were analysed using the Wilcoxon test. The correlation between gene expression and clinical variables was assessed using Fisher’s exact test.

**Results:**

Transcriptomic analysis revealed the presence of 1,687 DEGs in PTC, and the expression of 804 and 883 genes was found to be upregulated and downregulated, respectively. *LAMB3* expression was significantly elevated in cancerous tissues when compared to that in paracancerous tissues (*p* < 0.001). *LAMB3* expression was significantly positively associated with PTC tumour tissue size (*p* = 0.001, *r* = 0.49) and T stage (*p* = 0.017, *r* = 0.339).

**Conclusions:**

Our findings suggest that *LAMB3* expression significantly increases in PTC and it is associated with tumor size and T stage.

**Supplementary Information:**

The online version contains supplementary material available at 10.1186/s12885-025-14916-0.

## Background

Thyroid carcinoma (TC) is the most common endocrine malignancy [1, 2]. In recent decades, its prevalence has rapidly increased to approximately 3.1% of all first-diagnosis cancers worldwide. Papillary thyroid carcinoma (PTC) is the most frequent TC, accounting for 80% of TC cases. Additionally, PTC has a 3-fold higher frequency in females than in males [3, 4]. PTC tends to metastasise to the lymph nodes; although the 5-year mortality rate of patients with PTC is approximately 98%, around 15% of patients experience recurrence and approximately 5% of patients experience a fatal outcome. Thyroid nodules are a prevalent clinical presentation and are found in around 20–76% of patients with PTC; however, only 7–15% of cases are malignant [5, 6]. Therefore, it is challenging to distinguish malignant tumours from thyroid nodules. Although fine needle aspiration cytology has become one of the most dependable diagnostic techniques for thyroid malignancies in recent years, its results are unsatisfactory in 10–15% of cases.

Currently, PTC is treated primarily with surgery and adjuvant thyroid hormone or radioiodine therapies [7], with typically good outcomes. However, 15% of patients experience postoperative recurrence. Therefore, better prognostic markers are required for patient prognosis and the selection of optimal treatment strategies. Clinical features including age, tissue type, tumour size, local invasion, and lymph node and distant metastases are commonly used as prognostic factors; however, these features cannot accurately predict the prognosis of individual patients. Identifying patients eligible for surgery, adjuvant therapies, or clinical follow-up requires an understanding of the molecular features of tumours that increase the risk of undesired outcomes.

Tumour aggression and progression is a major contributor to poor clinical outcomes in PTC cases, which are closely related to the extracellular matrix (ECM) and basement membrane destruction. which Laminin-332 is an ECM protein produced by human keratin-forming cells. Laminin-332 is encoded by LAMA3, lam3, and LAMC2 and is involved in the aggressiveness of several cancers [8–11]. In particular, *LAMB3* participates in the infiltration and migration of pancreatic, lung, head and neck [12], prostate [13], and gastric [8] tumours. This gene affects cell adherence, multiplication, migration, and differentiation [14] and is associated with the clinicopathological characteristics of cancers [13, 15]. Upregulation of *LAMB3* has been observed in pancreatic ductal adenocarcinoma wherein it regulates cell cycle arrest and apoptosis, thus altering tumour behaviour in terms of multiplication, aggression, and metastasis by modulating the PI3K/Akt signalling pathway [16]. In primary lung cancers, *LAMB3* is upregulated; the high expression positively correlates with the grade of malignant differentiation, advanced tumour stage, lymphatic lung tumour metastasis, and the enhanced migration and infiltration of lung cancer cell lines [17]. Additionally, *LAMB3* reportedly promotes gastric cancer via aberrant positive regulation of promoter demethylation [8].

The development of full-length transcriptome sequencing technology has allowed the analysis of DEGs to improve our understanding of PTC. Yet, there are a few applications of full-length transcriptome sequencing in PTC diagnosis and treatment. In this study, we employed full-length transcriptome analysis to screen genes that are closely associated with PTC occurrence and progression, with the aim of identifying biomarkers for PTC diagnosis and therapy.

## Methods

### Patients

Each study participant voluntarily provided informed consent. The criteria for inclusion were as follows: (i) 18 to 80 years of age with positive diagnosis of PTC using paraffin-embedded biopsy samples; (ii) no prior thyroidectomy; (iii) primary cancer without previously receiving chemotherapy, radiotherapy, targeted therapy, cytokine therapy, or immunotherapy; or (iv) no history of neck radiation. The criteria for exclusion were as follows: (i) prior radiation to the neck or a history of radiotherapy or chemotherapy; (ii) thyroid cancer surgery; or (iii) another primary malignancy.

### Sample collection

Pairs of fresh tissues were obtained from 15 participants with PTC diagnosed from September 2021 to May 2022 at the Thyroid Surgery of The First Attached Hospital of Jinzhou Medical University. Cancerous and paracancerous (2 mm distal to the tumour site) tissues were obtained, instantly cryopreserved in liquid nitrogen, and shipped to Biomarker Biotechnologies Co. Ltd. (Beijing, China) for RNA sequencing.

### RNA extraction and cDNA Preparation

TRIzol reagent (Takara Bio Inc., Kyoto, Japan) was used to extract the RNA. The mRNA portion of the total RNA was assessed using the Implen NanoPhotometer spectrophotometer (Implen, Westlake Village, CA, USA). RNA was reverse transcribed to cDNA and these cDNA libraries were built from total RNA using the cDNA-PCR Sequencing Kit (SQK-PCS109; Oxford Nanopore Technologies [ONT], Oxford, UK). Fourteen cycles of PCR amplification were conducted using LongAmp Tag DNA Polymerase (New England Biolabs, Ipswich, MA, USA). The final cDNA libraries were analysed using FLO-MIN109 flow cells (R9 Version, FLO-PRO002; ONT) and the PromethION48 platform (ONT).

### Sequencing data and quality control

The initial format of the Nanopore sequencing data was second-generation FAST5. Guppy software (V6.4.6) was used to base-call the data. The fastq format was substituted for the original FAST5 format to analyse data quality. Clean data were checked against the database to further improve accuracy, and ribosomal RNA was filtered before further analysis. Comparison of whole-field sequences to reference genome sequences was performed using the min2 software (version 2.16; ONT), and consensus sequences were finally obtained using pinfish (v0.1.0; ONT). For each sample, concordant sequences were integrated and mapped to all reference genomes using minimap2, and the results were compared. Redundant sequences were removed, and sequences with an identity below 0.9 and coverage below 0.85 were removed. Results differing only at the 5′-end exon were integrated for variable splicing analyses. Redundant results from each sample were integrated to yield a nonredundant transcript sequence. The transcript structure was identified using whole-genome sequencing and compared with transcripts from the genome using GffCompare (version 0.9.8) to identify novel genes and transcripts.

### Analysis of differentially expressed genes (DEGs)

Gene expression was determined as counts per million (CPM): CPM = reads assigned to transcripts/total reads mapped to samples. Gene expression was compared between cancerous and paracancerous tissues in terms of fold change (FC). Genes with an FDR < 0.01 and |log2(FC)| ≥ 1 found by DESeq2 (version 1.6.3) were assigned as differentially expressed.

### Gene ontology (GO) analysis

GO analysis was employed to uncover significantly enriched terms considering biological processes, molecular functions, and cellular components; a *p-*value of < 0.05 was considered significant enrichment. R/clusterProfiler package was used for GO analysis.

### Kyoto encyclopaedia of genes and genomes (KEGG) analysis

The *q*-value is the *p*-value after correcting for multiple hypothesis tests, and the lower the *q*-value, the higher the confidence of significant enrichment for DEGs in a given pathway. The KEGG database (http://www.genome.jp/kegg) [18] was used for pathway annotation of DEGs. A *p* value of < 0.05 indicated significant enrichment.

### Immunohistochemistry staining of tissue microarrays

Tissue microarray analysis was approved by the Ethics Council of Shanghai Outdo Biotech Co., Ltd. (Ethics number: SHYJS-CP-1907007). Human tissue microarrays containing 58 pairs of cancerous and paracancerous tissues collected from patients with PTC were provided by Shanghai Outdo Biotech Co. Ltd. (Shanghai, China). The tissue chips were placed in an oven, maintained at 63 °C for 1 h, deparaffinised in xylene, and rehydrated in a graded ethanol series. After retrieving tissue antigens, the tissue sections were placed in distilled water at room temperature (20–26 °C) and allowed to cool for over 10 min. Subsequently, the tissue sections were rinsed thrice with phosphate-buffered saline with Tween for 1 min and then immersed in anti-*LAMB3* primary antibody (SC133178, 1:50 dilution, Mouse Monoclonal; Santa Cruz Biotechnology, Dallas, TX, USA) at 4 °C overnight. The sections were rinsed thrice with phosphate-buffered saline for 1 min. Thereafter, the sections were incubated with the appropriate secondary antibody (K8002; Dako, Glostrup, Denmark) for 10 min. The sections were rinsed thrice with phosphate-buffered saline for 1 min. The stain was developed by treating the sections with horseradish peroxidase for 15 min. The sections were stained with diaminobenzidine and counterstained with haematoxylin, for 1 min each.

### Interpretation of immunohistochemical staining

The scanner(Aperio XT, LEICA) was used for immunohistochemical analysis. Two experienced pathologists independently assessed the staining results. More than three fields of view with different staining intensities were selected, their positive rates were estimated, and the average positive rate was calculated. Staining intensity scores were as follows: 0 (negative), 0.5 (0.5+), 1 (1+), 2 (2+), and 3 (3+). A positive stain score was between 0% and 100%. The overall score was estimated as the product of “staining intensity score” and “stain positivity rate” (0–300%).

### Statistical analysis

The Wilcoxon method was used to determine whether protein expression differed significantly between the cancerous and paracancerous tissues. Fisher’s exact test was used for analysing the relationship between protein expression and clinical parameters. *p* < 0.05 indicated significance. R software (version 4.2.0; R Foundation for Statistical Computing, Vienna, Austria) was used for data analysis. Additionally, we assessed the clinicopathological parameters (age, sex, lymph node migration, tumour size, and T and TNM stages).

## Results

### Analyses of DEGs

**Fig. 1:**
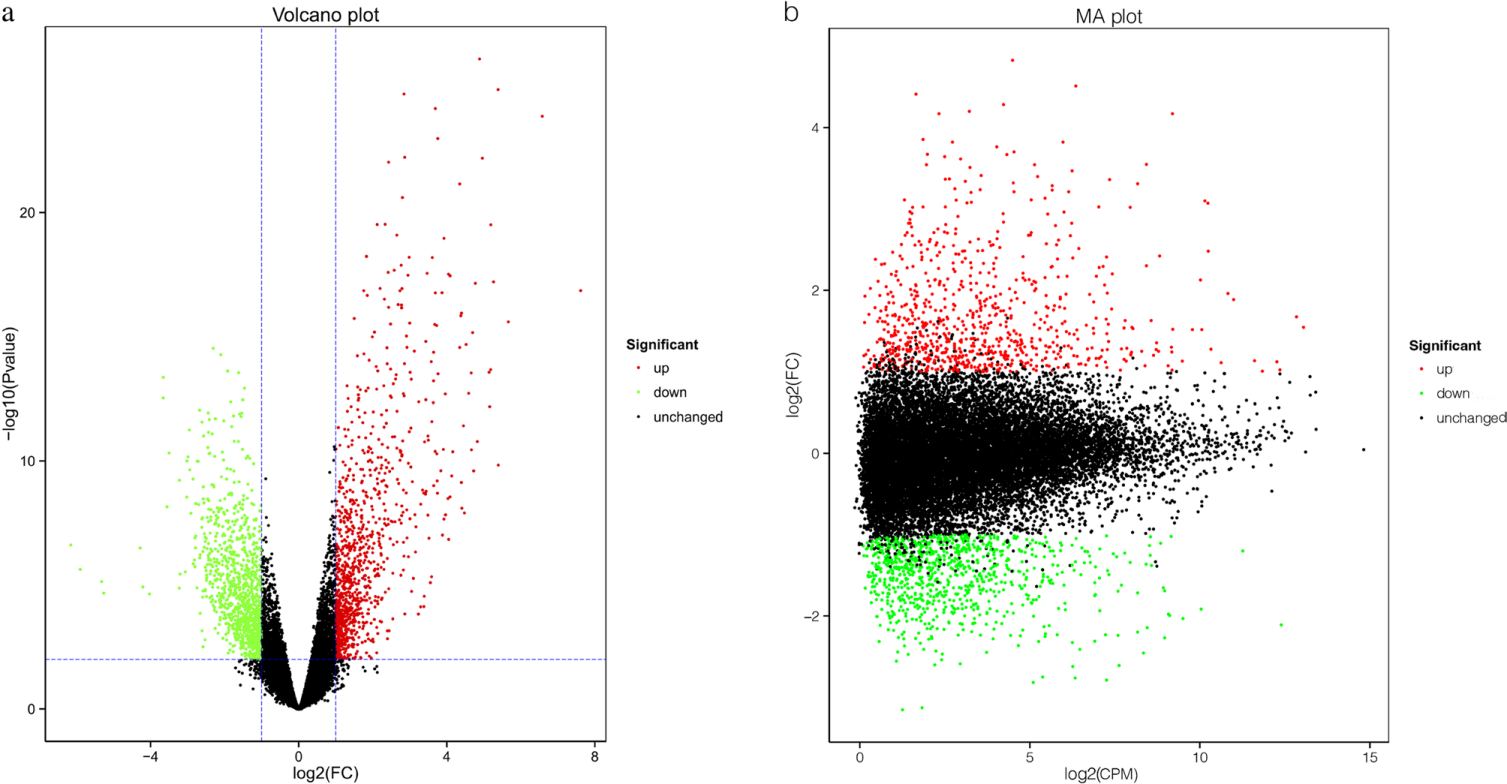
Differential gene expression analysis of 15 paired papillary thyroid carcinoma (PTC) and paracancerous tissues. **a** Volcano plot of differentially expressed genes (DEGs). The abscissa represents a log2 fold change (FC) for each gene and the ordinate represents a *p*-value for log10 log-transformed analysis of significance. The upregulated DEGs are represented by red dots, downregulated DEGs by green dots, and genes with no change in expression by black dots. **b** Bland–Altman plot of DEGs. Each point in the DEG plot indicates one gene. The *x*-axis is the A value: log2 count per million (CPM), which is the log of the mean value of expression in the two specimens. The *y*-axis is the M value: log2(FC), that is, the log of the fold difference in gene expression between the specimens

We identified 1,687 DEGs; among them, 804 were upregulated and 883 were downregulated (Table 1) (additional file 1). The volcano plot of DEGs between the cancerous and paracancerous tissue samples has been shown in Fig. 1a. The Bland–Altman plot of DEGs has been shown in Fig. 1b.

**Table 1:** Number of DEGs between 15 pairs of cancerous and paracancerous tissues from patients with papillary thyroid carcinoma

DEG set	Number of DEGs	Upregulated	Downregulated
N vs. C	1687	804	883
N1 vs. C1	929	427	502
N2 vs. C2	897	522	375
N3 vs. C3	474	360	114
N4 vs. C4	882	397	485
N5 vs. C5	639	382	257
N6 vs. C6	990	497	493
N7 vs. C7	490	114	376
N8 vs. C8	875	485	390
N9 vs. C9	1437	469	968
N10 vs. C10	825	603	222
N11 vs. C11	892	550	342
N12 vs. C12	380	316	64
N13 vs. C13	826	600	226
N14 vs. C14	452	353	99
N15 vs. C15	156	106	50

*DEG* Differentially expressed gene

### Top 50 upregulated DEGs and selection of *LAMB3*

**Fig. 2:**
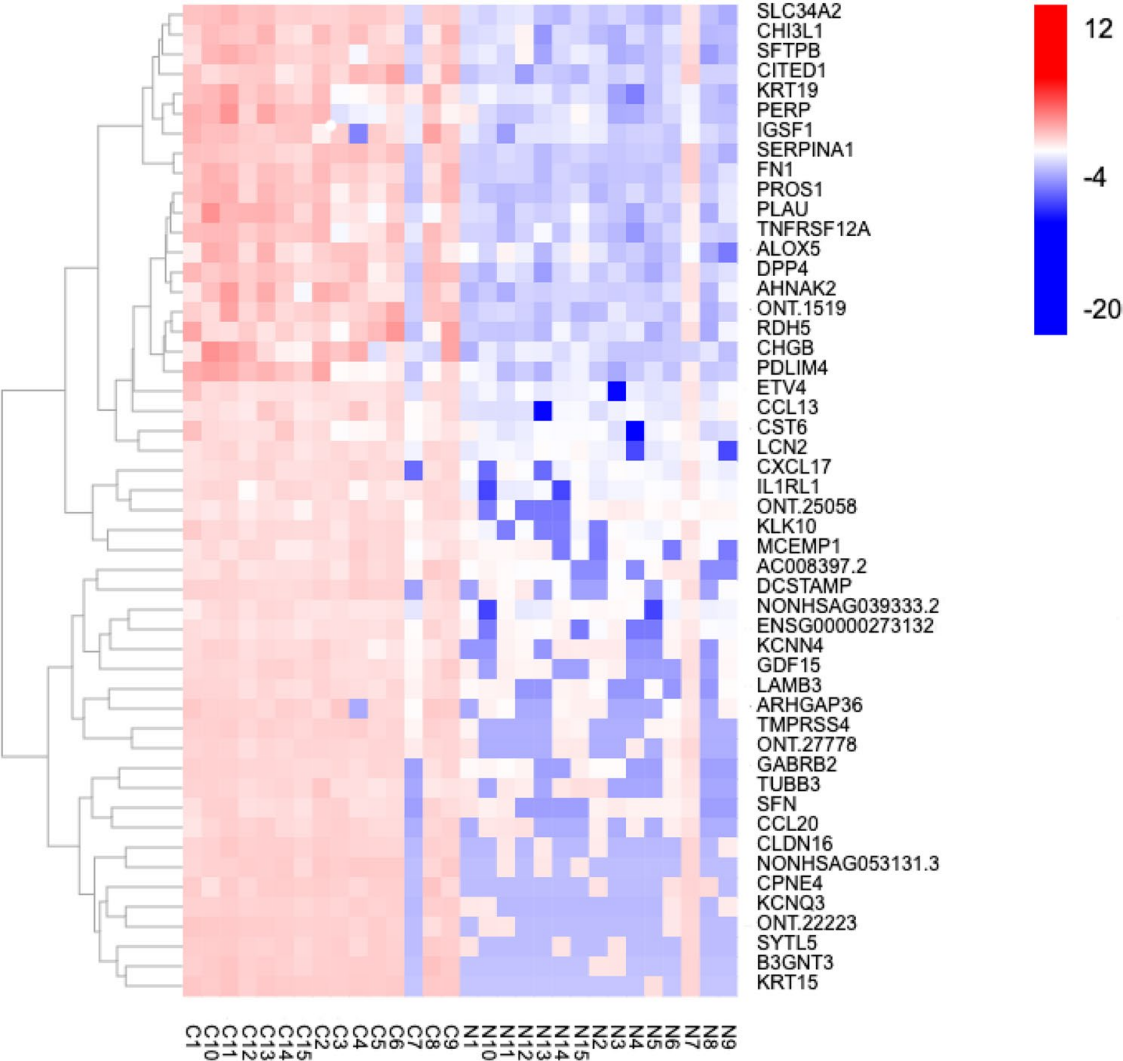
Clustering heat map of the top 50 upregulated DEGs in 15 pairs of PTC samples. The specimen names are represented by the *x*-axis, and the DEGs are represented by the *y*-axis. Upregulation is represented by red, downregulation by blue, and no difference in gene expression by white

**Fig. 3:**
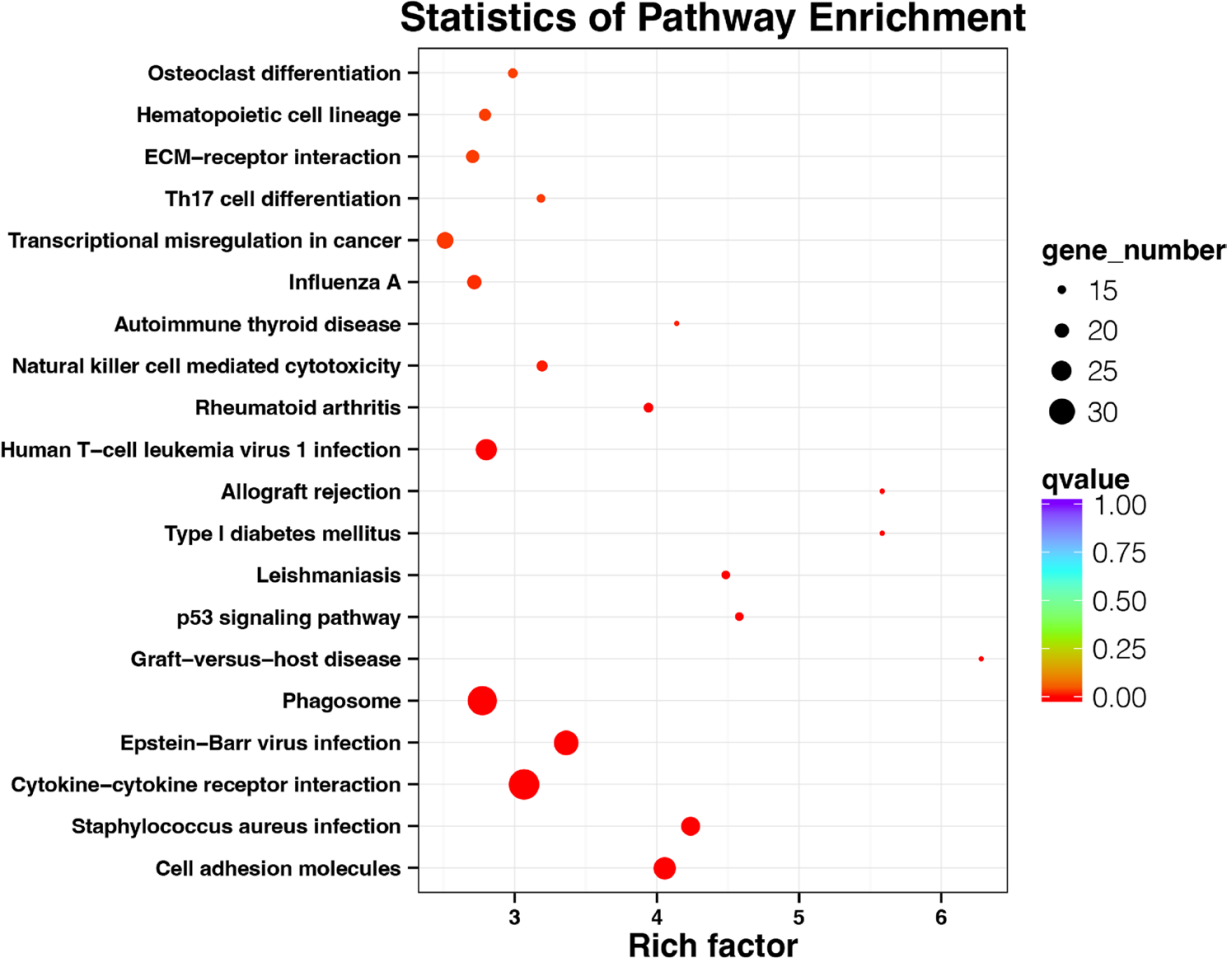
Top 20 Kyoto Encyclopaedia of Genes and Genomes (KEGG) pathways enriched by DEGs. The plot magnitude represents the count of DEGs in that pathway, and the colour represents the degree of enrichment. The *y*-axis is the enriched factor, and the greater the value, the stronger the enrichment

Figure 2 presents the 50 most significantly upregulated genes. By analyzing the up-regulated Top50 genes and Top20 enriched pathways(Fig. 3), we focused on ECM-recepor interaction in Top20 enriched pathways(Fig. 3). We identified the Top50-enriched gene *LAMB3* enriched in the ECM-recepor interaction pathway as the candidated gene of the study (Fig. 5).

### KEGG pathway enrichment analysis of the upregulated DEGs

The KEGG pathway results for the 804 upregulated genes revealed the top 20 enriched pathways were associated with cell adhesion molecules, *Staphylococcus aureus* infection, and Epstein–Barr virus infection (Fig. 3).

### GO term enrichment analysis of the upregulated DEGs

**Fig. 4:**
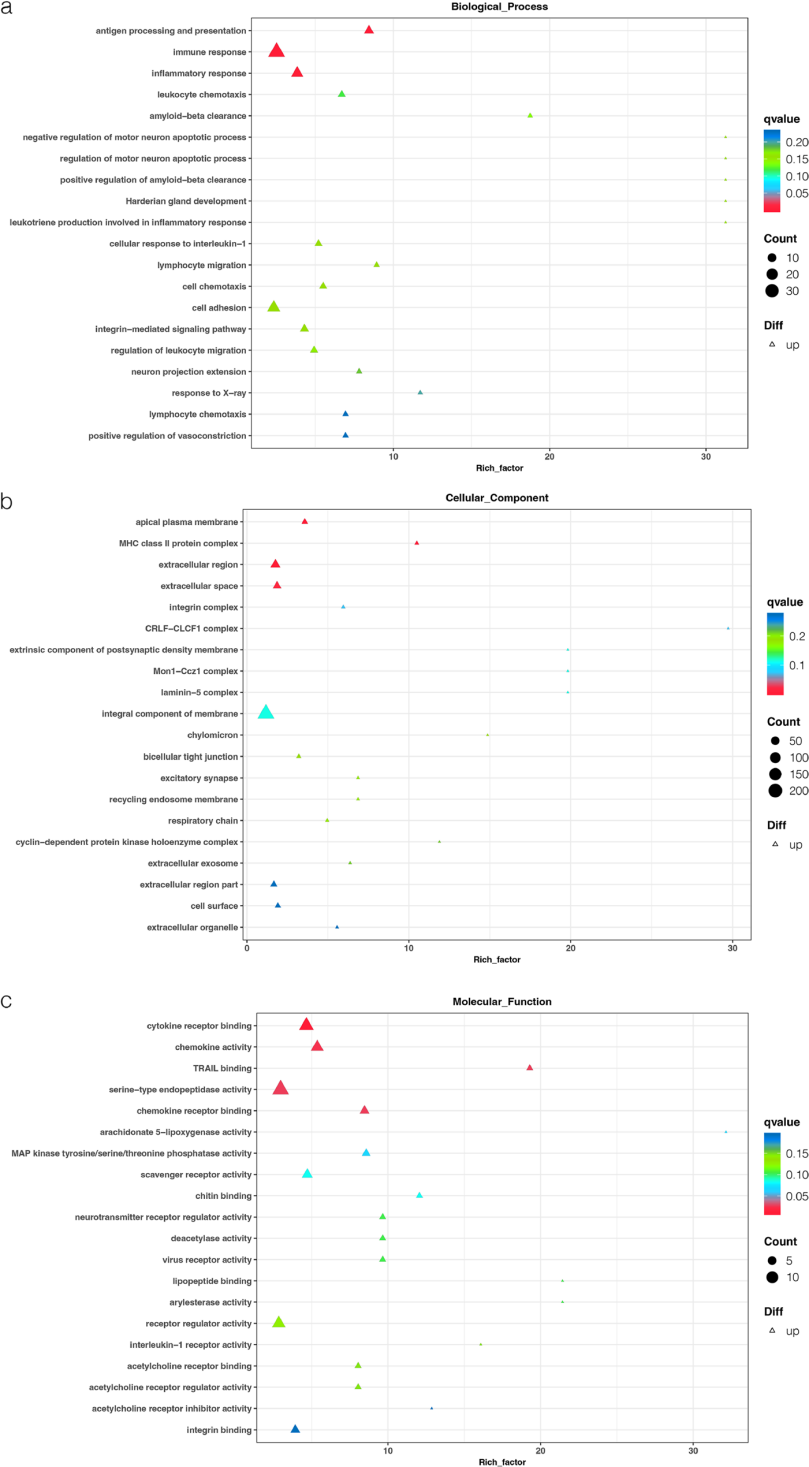
Analysis of the top 20 Gene Ontology (GO) terms enriched by the upregulated DEGs. The vertical coordinate includes the names of the enriched terms, and the abscissa denotes the enrichment factor. The magnitude of the point denotes the quantity of enriched genes, that is, the bigger the point, the greater the genes are enriched for that item. The colour of the point indicates the *q*-value, which is the *p*-value after adjustment for multiple assumption testing. The lower the *q*-value, the more credible the enrichment of DEGs for the given term. A, biological process (BP); B, cellular component (CC); C, molecular function (MF)

Figure 4 presents the top 20 GO enriched terms for the 883 upregulated DEGs. Significantly enriched biological process terms included antigen processing and presentation, immune response, and inflammatory response. Significantly enriched cellular component terms included MHC class II protein complex, apical plasma membrane, and extracellular region. Molecular function terms included cytokine receptor binding, chemokine activity, and chemokine receptor binding.

### GO term enrichment analysis of *LAMB3*

Table 2 reveals the enriched GO terms for *LAMB3*.

**Table 2:** Enriched GO terms for *LAMB3*

Category	ID	Description	*p*	q
BP	GO:0050873	Brown fat cell differentiation	0.002	0.30
MF	GO:0044877	Macromolecular complex binding	0.061	0.47
CC	GO:0005610	Laminin-5 complex	0.041	0.70

*BP* Biological process; *CC* Cellular component; *MF* Molecular function; *GO* Gene Ontology

### KEGG pathway enrichment analysis of *LAMB3*

**Fig. 5:**
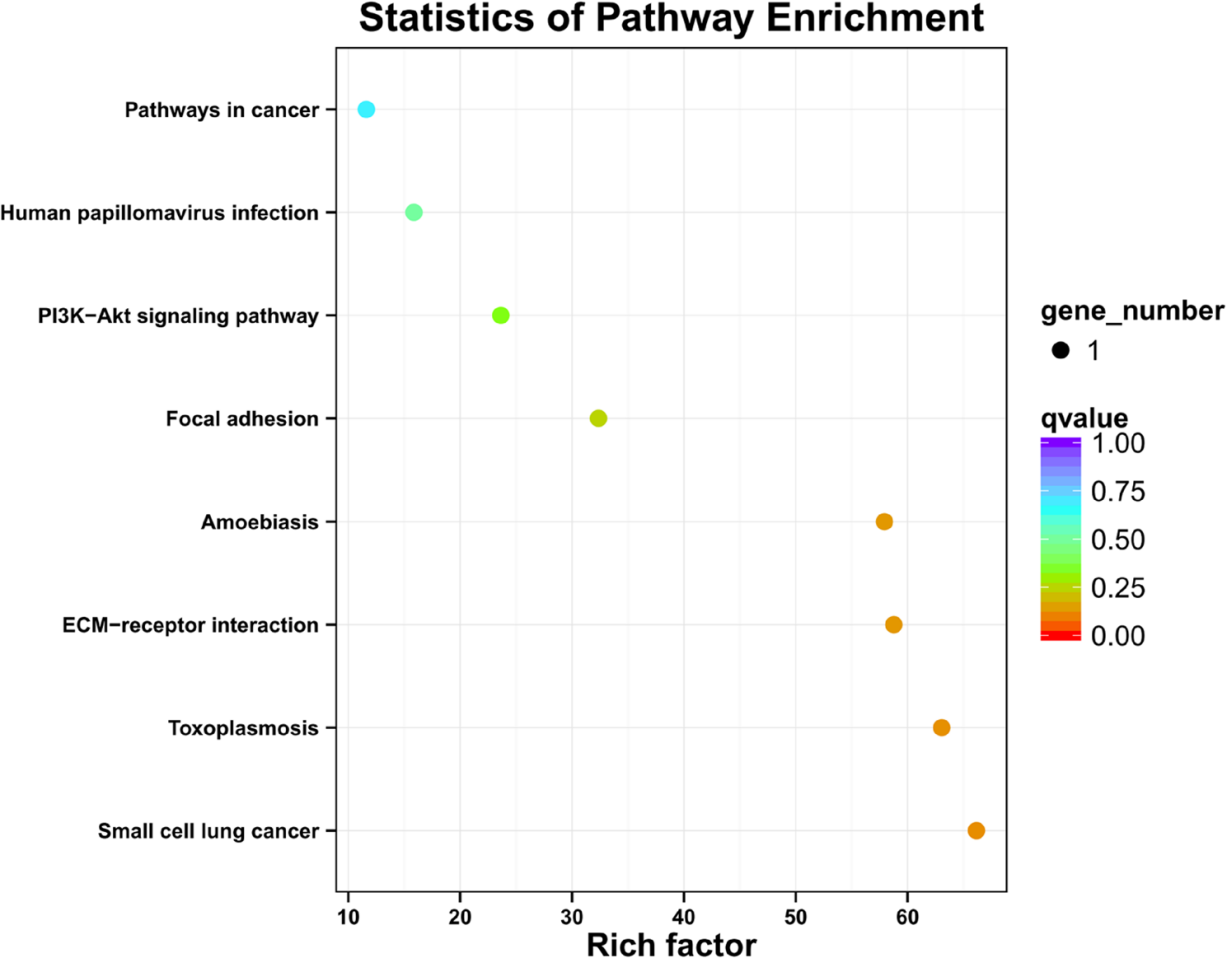
KEGG pathway enrichment analysis of *LAMB3*. The lower the *q*-value, the more accurate the enrichment of *LAMB3* for the given pathway

Figure 5 reveals the KEGG pathway enrichment analysis results for *LAMB3.* The enriched pathways included the ECM-recepor interaction pathway and toxoplasmosis. The KEGG analysis suggested that *LAMB3* was mainly enriched in the ECM-recepor interaction pathway, and *LAMB3* was selected from the 50 most significantly upregulated genes for further investigation.

### *LAMB3* expression in PTC tissue microarrays

**Fig. 6:**
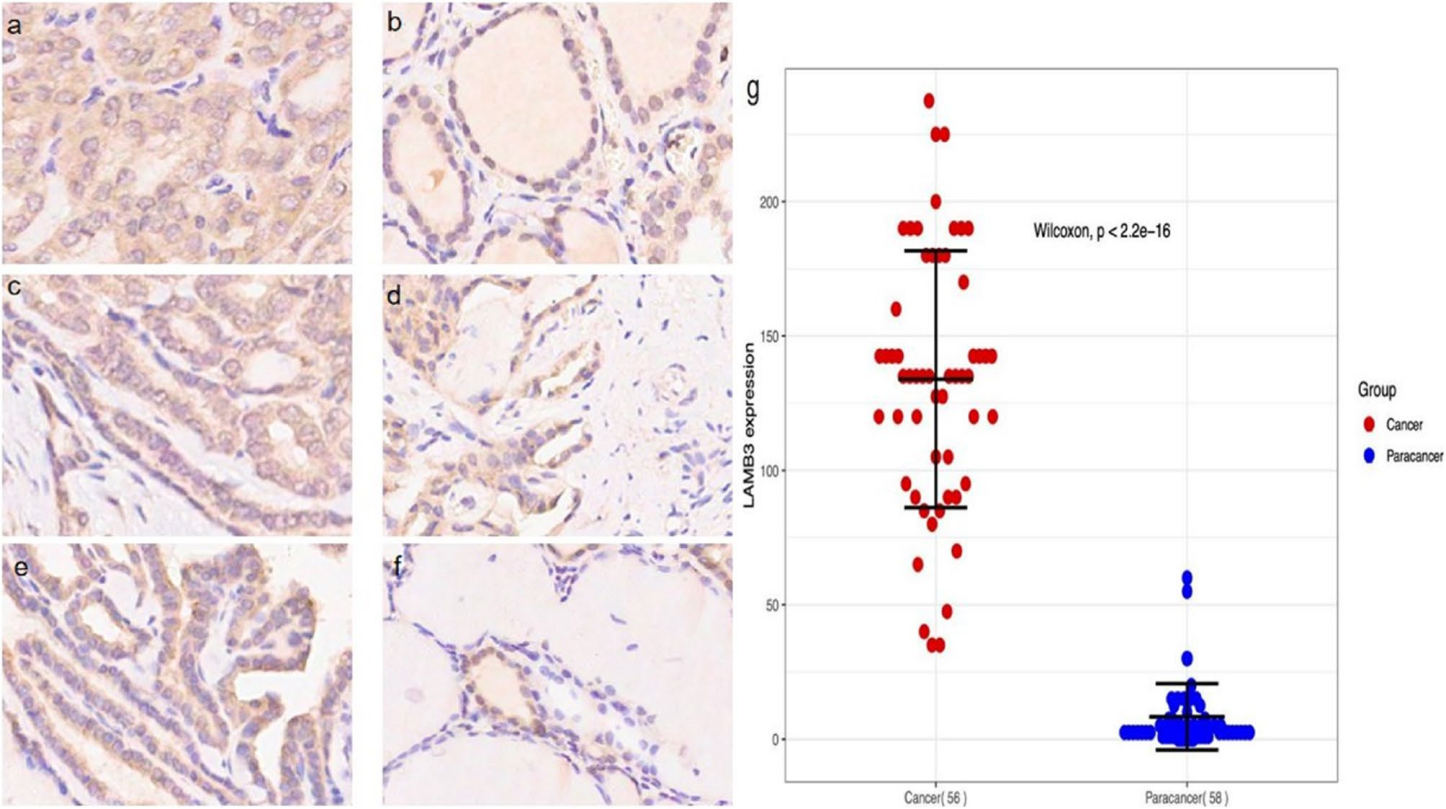
Representative images of different scores of immunohistochemical staining for *LAMB3* in PTC tissue microarrays. Immunohistochemical score: Cancerous tissue: a 190%; c 112.5%; e 52.5%. Paracancerous tissue: b 75%; d 37.5%; f 7.5%.(magnification: ×200). Brown represents *LAMB3* staining and blue represents haematoxylin staining. g *LAMB3* expression in PTC cancerous and paracancerous tissues

Immunohistochemical staining revealed that the *LAMB3* expression was substantially elevated in cancerous tissues relative to that in paracancerous tissues (*p* < 0.001; Fig. 6a–g). Figure 6 shows representative *LAMB3* staining in PTC tissue microarrays. The images of the PTC tissue microarrays for *LAMB3* immunohistochemical staining were shown in additional file 2. The formula for calculating the expression index when evaluating the results of immunohistochemistry was shown in additional file 3.

### Correlation between *LAMB3* expression and clinicopathological parameters in PTC

*LAMB3* expression in PTC significantly correlated with tumour size (*p* = 0.001, *r* = 0.49) and T-stage (*p* = 0.017, *r* = 0.339), but not with other clinicopathological factors (Table 3). *LAMB3* expression was higher in cancerous tissues > 2 cm than in those ≤ 2 cm in size. Furthermore, its expression was higher in T2–T4 stages than in T1 stage.

**Table 3:** Correlation between *LAMB3* expression and clinicopathological characteristics

Clinicopathological factor	Variable	*LAMB3* expression		Total	*p*	*r*
		Low	High			
Sex					0.55	0.104
	Male	17	5	22		
	Female	23	11	34		
Age (years)					0.558	0.102
	≤ 48	22	7	29		
	> 48	18	9	27		
Tumour size					0.001	0.49
	≤ 2 cm	31	4	35		
	> 2 cm	9	12	21		
Tumour number					1	−0.032
	Single	34	14	48		
	Multiple	6	2	8		
T-stage					0.017	0.339
	T1	25	4	29		
	T2–T4	15	12	27		
N-stage					1	−0.022
	N0	21	9	30		
	N1	18	7	25		
TNM-stage					0.763	−0.059
	I/II	25	11	36		
	III/IV	15	5	20		

*T-stage* Tumour stage; *N-stage* Node stage; *TNM-stage* Tumour node metastasis stage

Immunohistochemical score: High >142.5%; Low ≤ 142.5%

## Discussion

In our study, we used full-length transcriptome sequencing to analyse DEGs in 15 pairs of cancerous and paracancerous PTC tissues. A total of 1,687 DEGs were identified, of those, 804 were upregulated and 883 were downregulated. Significant differences in *LAMB3* expression were observed in cancer tissues compared with paracancerous tissues. According to the results of the differential expression of genes and KEGG analysis, we further investigated *LAMB3* as a candidate biomarker among the upregulated genes. To our knowledge, this research was the first to examine, at the protein level, the correlation between *LAMB3* expression and clinical features detected by immunohistochemistry. *LAMB3* immunohistochemistry expression of PTC tissue microarrays was substantially elevated in cancerous tissues relative to that in paracancerous tissues, which corresponded with our RNA-sequencing data. These findings indicate the suitability of the considerable differential *LAMB3* expression in cancerous tissues as a biomarker for PTC.

Laminins are an integral element of the basement membrane and are required for various critical biological processes [19], including wound repair, tissue formation, and pathological events, such as tumorigenesis [20]. *LAMB3* belongs to the laminin large extracellular glycoprotein family. Our findings correspond with those of previous studies demonstrating the upregulated expression of *LAMB3* in PTC [21]. Previous studies have also shown that *LAMB3* contributes to PTC metastasis [21]; however, this differs from the present findings, and the difference in the results may be attributed to the smaller population of this study. Furthermore, higher *LAMB3* expression was notably associated with larger and advanced tumour grade in this study, indicating a potential disruption of laminin expression in PTC, which maybe associated with disease invasiveness.

The current study did have certain limitations. We only preliminarily examined *LAMB3* protein expression in tissue and its functional significance. We did not comprehensively assess the diagnostic and prognostic value of *LAMB3* in PTC. In addition, the small sample size for immunohistochemistry leads to limited representativeness. Future research should focus on validating the current findings in larger samples and clinically accredited platform to assess the clinical significance of *LAMB3* expression in PTC.

## Conclusions

In this study, we revealed the expression profiles of PTC and the functional of differentially significantly expressed genes, and discovered potential biomarker *LAMB3* for the diagnosis and treatment of PTC. And we validated *LAMB3* upregulation via PTC tissue microarray analysis, where it was indeed associated with PTC tumor size and T stage. *LAMB3* expression thus hold potential as a clinical biomarker for PTC disease severity assessment.

## Supplementary Information

Additional file 1.

Additional file 2.

Additional file 3.

## Data Availability

Our database is stored in NCBI(PRJNA1033723).

## Abbreviations

DEG: Differentially expressed gene
ECM: Extracellular matrix
FC: Fold change
FDR: False discovery rate
GO: Gene Ontology
KEGG: Kyoto Encyclopaedia of Genes and Genomes
*LAMB3*: Laminin subunit beta-3
PTC: Papillary thyroid cancer
TNM: Tumour node metastasis
