# Supplementary figures and images for: Full-length transcriptome analysis of papillary thyroid carcinoma reveals correlation between LAMB3 expression and clinical features
Source: BMC Cancer. 2025 Oct 25;25:1646. doi: 10.1186/s12885-025-14916-0 (PMC12554243; doi:10.1186/s12885-025-14916-0)

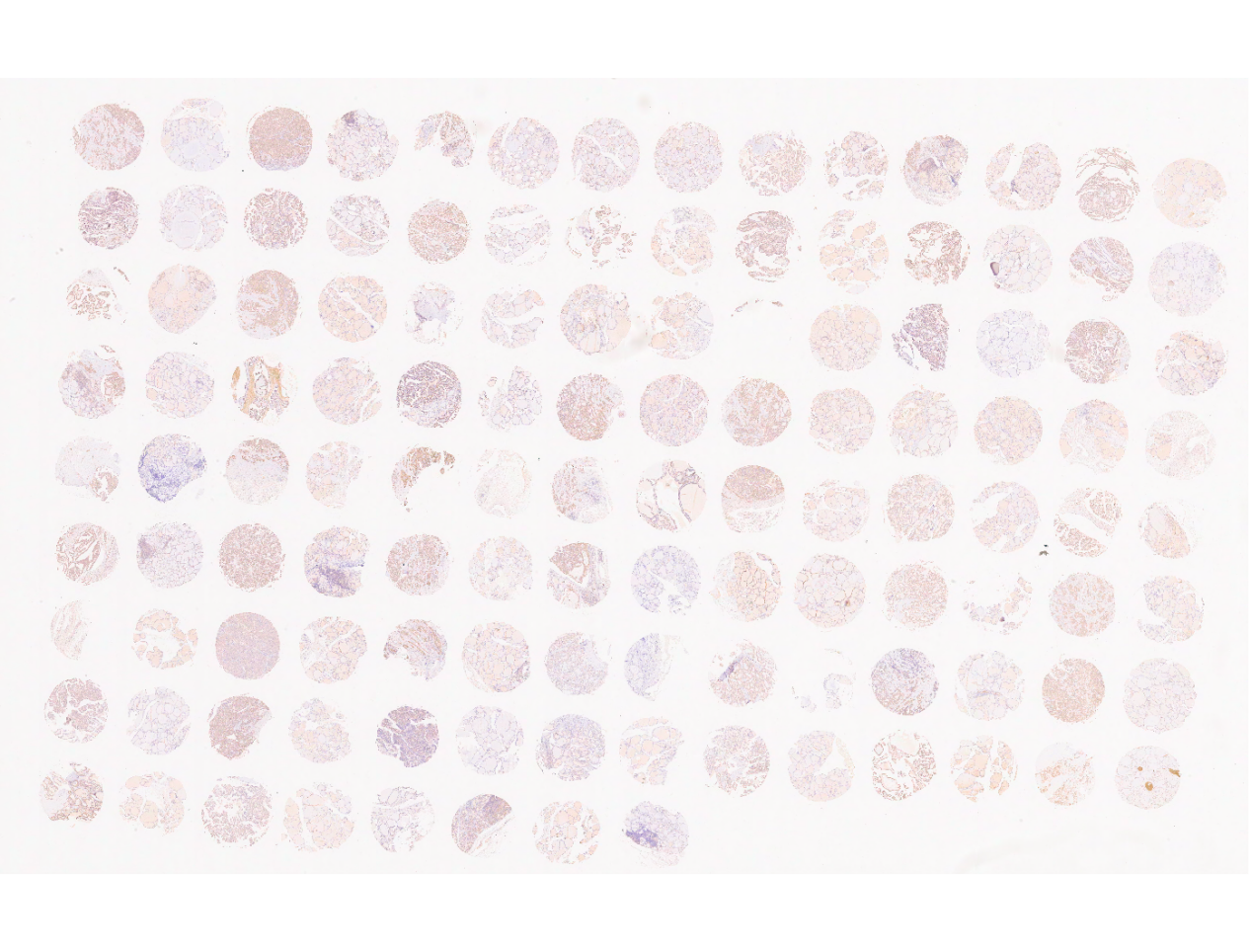

Supplement: Supplementary file 2 — Additional file 2. [file 12885_2025_14916_MOESM2_ESM.docx]
